# Highly sensitive SnO_2_ sensor via reactive laser-induced transfer

**DOI:** 10.1038/srep25144

**Published:** 2016-04-27

**Authors:** Alexandra Palla Papavlu, Thomas Mattle, Sandra Temmel, Ulrike Lehmann, Andreas Hintennach, Alain Grisel, Alexander Wokaun, Thomas Lippert

**Affiliations:** 1 Paul Scherrer Institut, Energy and Environment Research Department, 5232 Villigen- PSI, Switzerland; 2 National Institute for Lasers, Plasma, and Radiation Physics, Lasers Department, Atomistilor 409, 077125, Magurele, Romania; 3 Microsens SA, Rue de la Maladière 71c, CH-2002 Neuchâtel, Switzerland; 4 EPFL Innovation Park, Building D, CH-1015 Lausanne, Switzerland; 5 Department of Chemistry and Applied Biosciences, Laboratory of Inorganic Chemistry, ETH Zürich, CH-8093 Zürich, Switzerland

## Abstract

Gas sensors based on tin oxide (SnO_2_) and palladium doped SnO_2_ (Pd:SnO_2_) active materials are fabricated by a laser printing method, i.e. reactive laser-induced forward transfer (rLIFT). Thin films from tin based metal-complex precursors are prepared by spin coating and then laser transferred with high resolution onto sensor structures. The devices fabricated by rLIFT exhibit low ppm sensitivity towards ethanol and methane as well as good stability with respect to air, moisture, and time. Promising results are obtained by applying rLIFT to transfer metal-complex precursors onto uncoated commercial gas sensors. We could show that rLIFT onto commercial sensors is possible if the sensor structures are reinforced prior to printing. The rLIFT fabricated sensors show up to 4 times higher sensitivities then the commercial sensors (with inkjet printed SnO_2_). In addition, the selectivity towards CH_4_ of the Pd:SnO_2_ sensors is significantly enhanced compared to the pure SnO_2_ sensors. Our results indicate that the reactive laser transfer technique applied here represents an important technical step for the realization of improved gas detection systems with wide-ranging applications in environmental and health monitoring control.

Tin dioxide (SnO_2_) is an n-type semiconductor with a bandgap of 3.6 eV, and used in a large range of applications, i.e. catalysis^1^, photovoltaic devices^2^, and rechargeable lithium batteries^3^. An important application of SnO_2_ is for the development of solid state devices, i.e. gas sensors based on thin porous films or nanoparticles^4^,^5^. Gas sensing by metal-oxide-semiconductors such as SnO_2_ is based on the reduction-oxidation reactions of the analyte which takes place at the surface of the semiconductor, which leads to a change in the sensor resistance. So far, various techniques have been developed to prepare SnO_2_ films and nanoparticles with different morphologies, crystallinity, and structure, such as sputtering^6^, laser ablation^7^, sol-gel^8^, or thermal evaporation^9^. However, these methods present some disadvantages, for example, the main disadvantage of sputtering is that the sensor surface has to be patterned with a photoresist and a consecutive lift-off process. The lift-off process limits the deposition temperatures that can be reached, due to possible decomposition of the photoresist. In addition, most of these conventional deposition techniques are not used in industrial commercial processes. Therefore, there is a continuous need for better sensors, that are more sensitive, selective, stable, and cheaper, which drives new developments in downsizing the sensing elements. In addition, downsizing the sensing elements and their application in micro and nano-devices determines the need for roll-to-roll additive techniques. Inkjet printing is a low cost process which is currently used for gas sensor coating. However, the main disadvantage of this technique is clogging of the nozzles, and the need of tailored inks, consisting of a solvent, powder, binder, and dispersants.

Therefore, improvements in the sensor materials deposition methods would expand the possibilities. In fact, laser based deposition techniques, instead of the well-established technologies such as sputtering or inkjet printing, represent an extremely versatile and flexible tool, suitable to produce thin and homogenous layers of different materials with lateral resolution in the micron scale (“pixels”). In particular, conventional laser-induced forward transfer (LIFT) is a simple process, where a laser beam is focused through a transparent support plate onto a precursor thin film of the material to be transferred. Every single pulse promotes the transfer of the thin film material onto a substrate that is usually placed parallel and facing the thin film at very short distances (<100 μm). LIFT is a versatile method, which allows the transfer of solid^10^,^11^,^12^ or liquid phase materials^13^,^14^,^15^ with very high resolution, in air or vacuum, without contamination and a minimal consumption of material. LIFT is also a useful technique for the fabrication of functional devices, i.e. sensors, organic light emitting diodes, etc^16^,^17^,^18^. In order to improve the process efficiency, and to protect the materials to be transferred from direct laser irradiation, different approaches to LIFT have been proposed. For example, in^14^ the authors add an intermediate metal layer to transfer DNA microarrays and in^19^ the transfer of a conductive polymers i.e. PEDOT:PSS with an Au interlayer is presented. Other examples include the addition of intermediate polymer layers (triazene or polyimide polymers) for the fabrication of thin film transistors or electroluminescent devices^20^.

In this work, a new approach, i.e. reactive LIFT (rLIFT) has been evaluated as an alternative method to LIFT, which allows the integration of SnO_2_ and Pd:SnO_2_ patterns for their use in micro-sensors. In rLIFT, the donors are UV absorbing metal complex precursors (i.e. metal acetylacetonates) which upon absorption of the laser light partially decompose to SnO_2_ or Pd:SnO_2_^21^. Solution based precursors are in particular appealing due to their low deposition (growing) temperatures which make them suitable for flexible substrates, and good potential for scaling-up. During the past decade, a large number of metal oxides have been prepared from metal acetylacetonates, for example TiO_2_, In_2_O_3_, SnO_2_^22^,^23^ and more recently ZnO nanowires^24^. rLIFT is a unique technique which combines the advantages of the low decomposition temperatures of the acetylacetonates together with the spatial resolution of the laser based method. The transformation of the metal precursor to SnO_2_ takes place partially during laser transfer as a result of the photochemical and thermal processes, which in this particular case are an advantage as they shorten the fabrication process. Therefore, the results presented in this work prove that rLIFT has great potential for the integration of active materials into micro-sensing devices.

## Results and Discussion

### LIFT printing of SnO_2_ precursor

**Figure 1:**
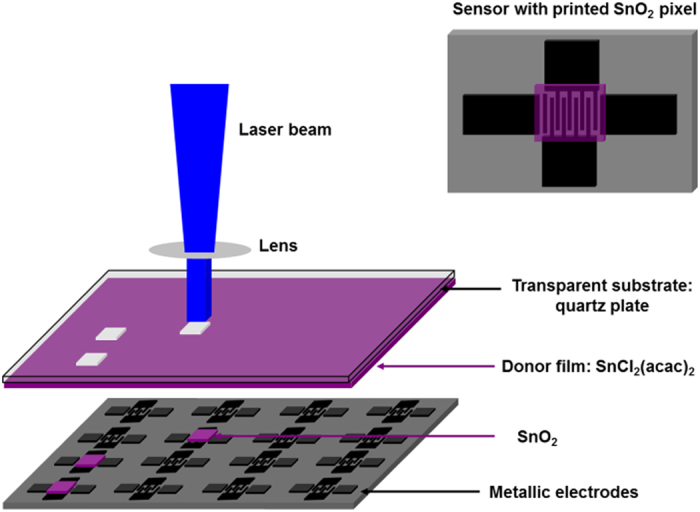
Scheme of the reactive laser-induced forward transfer process.

Reactive laser-induced forward transfer (see Fig. 1 for a scheme of rLIFT) is a useful process which combines the advantages of an additive technique with the possibility of decomposing materials partially upon exposure to laser light. rLIFT doesn’t require the use of an absorbing (sacrificial) layer, which in turn lowers the risk of printed material contamination, and the acetylacetonates decompose into small gaseous species. This work is focused on the fabrication of highly sensitive SnO_2_ and Pd doped SnO_2_ sensors by reactive LIFT and the evaluation of the performances of the rLIFT-ed sensors towards different analytes. Therefore, we shall only briefly describe the parameter optimization for laser transferring functional SnO_2_ pixels.

Previous studies have shown that by a careful choice of the transfer parameters, regular, well defined SnO_2_ pixels can be printed onto glass substrates from donor films of metal complex precursors^21^. The laser fluence is an important parameter to achieve a regular, debris free transfer. According to previous studies^21^, the range of laser fluences for which successful transfers are obtained, i.e. SnO_2_ pixels with a surface structure comparable to the donor film, is 200–400 mJ/cm^2^. The metal complex precursor donor film is about 1 μm thick, while the transferred pixels are in the range 900 nm–1 μm thick, as determined by measuring the height of the transferred pixels with a profilometer. Transfers onto IDT structures do not show significant differences from the transfers onto glass receivers. The edge quality of the pixels and their morphology is similar to those of pixels transferred onto glass. Therefore, based on previous experiments, the pixels used for the fabrication of sensors (in this work) are transferred at 300 mJ/cm^2^ laser fluence and are around 900 nm thick.

### Base resistance in dry and humid environment

The base resistance and the sensitivity towards C_2_H_5_OH are tested in dry and in humid atmosphere to simulate the influence of different environments which will be encountered in real applications. SnO_2_ is in general able to detect C_2_H_5_OH in the low ppm range and at comparable low temperatures (300 °C)^25^. C_2_H_5_OH gas sensors are particularly important as they can be applied in many fields, such as control of fermentation processes or to monitor drunken drivers. To test the sensitivity, the sensor-like structures are first heated until a stable temperature of 350 °C is reached.

**Figure 2:**
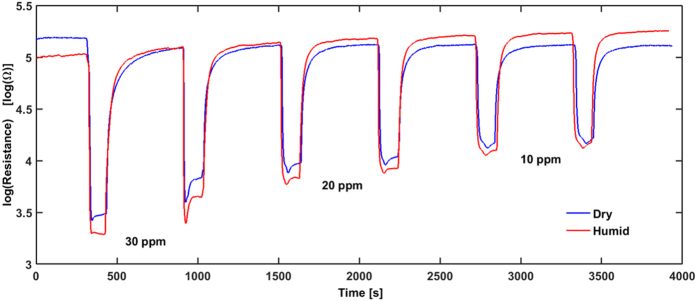
Response curves (measured at 350 °C) of the laser printed SnO_2_ sensor to different concentrations of ethanol in dry (blue curve) and humid (red curve) environment. Each concentration is applied twice for 2 minutes and the sensor recovered after 8 minutes its initial baseline.

The sensors are kept at a constant temperature until the base resistance is stable prior to exposure to air containing C_2_H_5_OH in the ppm range. After 2 minutes the C_2_H_5_OH flow is switched off and the SnO_2_ recovers its initial resistance within 8 min before the next injection of analyte takes place. A typical sensing curve can be seen in Fig. 2 with concentration of 30, 20, and 10 ppm, each applied twice in consecutive order.

The recovery of the base resistance after C_2_H_5_OH injections is in general fast and the drift over 1 h is small, resulting in a small deviation of R_0_ for individual samples. The same behavior can be noticed for the sensors measured in humid SA.

### Sensing C_2_H_5_OH and CH_4_

**Figure 3:**
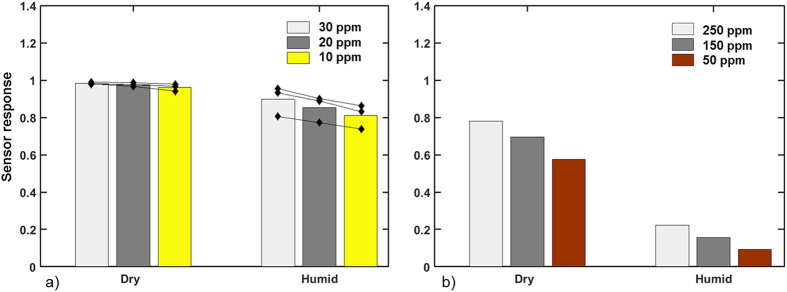
Sensor response in dry and humid environment of rLIFT fabricated SnO_2_ sensors exposed to different concentrations of (**a**) ethanol (at 350 °C) and (**b**) methane (at 500 °C).

The results of detecting C_2_H_5_OH vapor in the low ppm range (30, 20, and 10 ppm) are shown in Fig. 3 for dry as well as for humid SA. The bars indicate the average behavior and the black lines are the individual samples. The response of the sensors is calculated as (R_air_− R_analyte_)/R_air_, where R_air_ is the electrical resistance of the sensors when exposed to (dry or humid) SA, and R_analyte_ is the electrical resistance of the sensor when exposed to the ethanol and/or methane analytes. The standard error of the mean sensor response is between ±1% and ±5%.

In dry SA (see Fig. 3a), samples show a response higher than 0.9 for all measured concentrations suggesting that saturation is already reached. The signal difference between the different concentrations is small, but still visible.

In (Fig. 3a) it is shown that the rLIFT printed SnO_2_ work rather well and all the printed sensors exhibit a high response even for 10 ppm of C_2_H_5_OH. Carrying out the same measurements in humid air represents a different picture. In general, all analyte responses are lower in humid air than in dry air^26^, even though the base resistance is not influenced much and the gradation between individual samples tends to be larger.

The rLIFTed sensor pads are also tested for their ability to detect CH_4_. CH_4_ is a highly volatile gas with a low explosion limit of 4.9% and a high explosion limit of 15.4%^27^. For detecting CH_4_ higher sensor temperatures (of 500 °C) are required as CH_4_ is more difficult to oxidize. Commercial sensors detecting CH_4_ work also at these higher temperatures.

Analyzing the sensitivities towards methane (see Fig. 3b) shows that the detection limit is as expected higher compared to C_2_H_5_OH and the sensitivity is far away from saturation (S = 1). The difference between the individual concentrations is clearly distinguishable for all measured samples. Switching from dry to humid air shows a different behavior than for the measurements with C_2_H_5_OH, i.e. the sensitivity measured in humid atmosphere is much lower than for dry air. This effect is not yet fully understood, however, it has been reported previously^28^,^29^,^30^ for sensors based on pure SnO_2_ measured in a wet atmosphere. A limited sensor response in a humid atmosphere can partially be expected by additional cooling of the SnO_2_ surface due to water related species (OH-groups). In^28^,^29^,^30^ the authors experimentally demonstrate (by Diffuse Reflectance Fourier Transformed Spectroscopy) that in the case of undoped SnO_2_ sensing layers, water competes with reducing gases for the presorbed oxygen species, the SnO_2_ sensors having a lower response in a humid environment.

### Drop casting the metal-complex precursor and its sensing abilities

**Figure 4:**
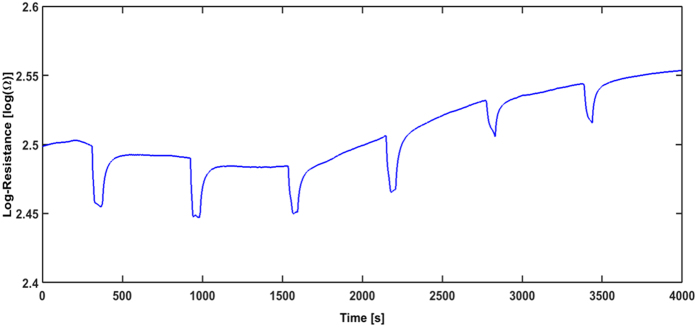
Response curve of a drop-casted SnCl_2_(acac)_2_ sample when exposed to ethanol (measured at 350 °C). The ethanol concentrations (from left to right) to which the sensor is exposed to, are 50 ppm, 30 ppm, and 10 ppm. Each concentration is applied twice.

Sensor pads are also prepared by drop casting to prove that the laser transfer improves the sensitivities reached by the sensor systems. The SnCl_2_(acac)_2_ solution is drop casted with a pipette onto the sensor pad before conditioning the sample with the standard thermal annealing procedure. The sensing characteristics and the base resistances are measured and the results are shown in Fig. 4. The base resistance is two orders of magnitude lower than for rLIFT printed samples which is due to the thicker SnO_2_ layers (i.e. 5 μm) obtained by drop casting^31^. Drop casting leads to a large material deposition on the IDT and therefore much more material contributes to the conductivity than in the case of LIFT printed SnO_2_. The drop casted samples show a similar R_0_ with a small drift after 1500 sec.

By exposing the film to C_2_H_5_OH clear signals for all the three measured concentrations are obtained. However, the analyte responses are much lower with S (50 ppm) = 0.2 than for the rLIFT printed sensors with S (30 ppm) = 0.9. Even for this low response there is still a clear signal for 10 ppm C_2_H_5_OH. A possible explanation for the improved performance of the rLIFTed SnO_2_ sensors as compared to the drop casted sensors could be due to the high velocities of the LIFT generated SnO_2_ pixels (around 200 m/s)^32^ which lead to better electrical contact between the electrodes and SnO_2_ pixels^33^.

### Long term stability of the rLIFTed sensors

A key aspect for commercial sensors is the long term stability of the sensing material. As SnO_2_ printed by rLIFT presents a new approach it is important to see if there is a possible problem with fast degradation of the transferred SnO_2_ pixels.

The samples are stored and tested towards different analytes in the laboratory under ambient conditions. After 1 year the same samples are measured again with the same parameters and the response and the base resistance are compared to the values acquired in the first round of measurements. This test is carried out to determine if storing the sensor for long time can destroy the sensing capability of the rLIFT-ed SnO_2_ layers.

**Figure 5:**
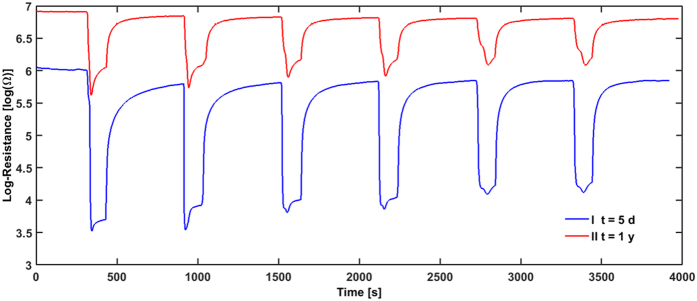
Response curve of a SnO_2_ sensor to ethanol (at 350 °C) measured 5 days after fabrication (blue curve) and the response curve of the same sensor measured 1 year later in the same test conditions. The ethanol concentrations (from left to right) to which the sensor is exposed to, are 30 ppm, 20 ppm, and 10 ppm. Each concentration is applied twice.

In Fig. 5 the sensing curves of a freshly prepared sample and the same sample measured after one year is shown. R_0_ changes by one order of magnitude for this sample (similar to commercial sensors). This drift of the base resistance could be due to the thermal effects arising from the heating-cooling cycles (during the operation time) which affect the pathway of current flowing through the SnO_2_ sensitive film^34^.

What is important is that the sensing characteristics are not changing, and the sensing curves exhibit still the same features like for the freshly prepared sensors. All concentrations are still clearly measurable for the stored samples. The signals towards C_2_H_5_OH injection appears smaller but this is mainly due to the logarithmic scale of the graph and the one order of magnitude higher R_0_. This shows clearly that for this sample the response is not affected much by a storage time of one year.

### Printing onto commercial sensors

**Figure 6:**
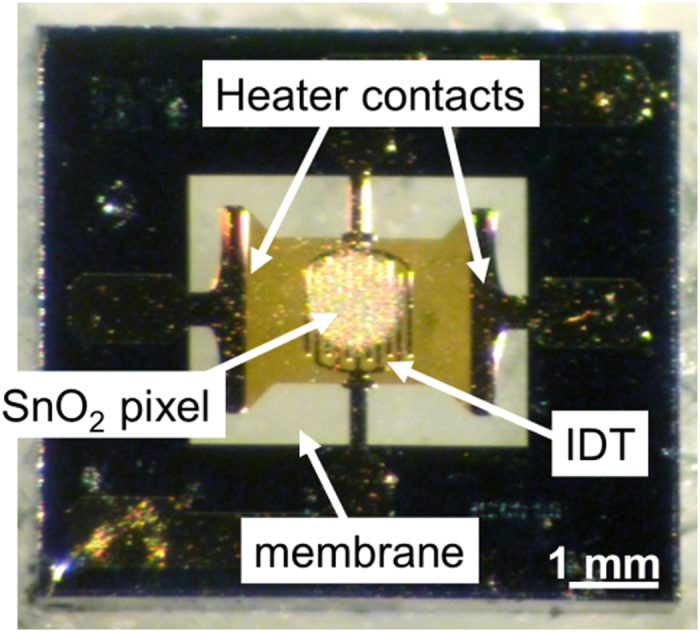
SnO_2_ rLIFTed pixel onto a commercial MSGS sensor structure. (**a**) A too large SnO_2_ pixel leads to a negligible resistance between the heater and the IDT. (**b**) A SnO_2_ pixel deposited only onto the IDTs. No measurable resistance between the heater and the IDTs occurs and the sensor is thus functional.

In order to prove the feasibility of LIFT for large scale technological applications, the transfer onto commercial sensor structures needs to be evaluated. The transfer of SnO_2_ pixels onto commercial sensors is more difficult to achieve, as the active area of the sensor is a free-standing membrane. During rLIFT the material to be transferred reaches high velocities, i.e. higher than 200 m/s, and the transferred pixels can damage the sensor receiver substrates. Therefore, in order to achieve a successful laser transfer onto such “delicate” structures, the sensors are reinforced, i.e. the sensors are backfilled with wax (as it is easily burnt off). In Fig. 6 an optical microscopy image of a SnO_2_ pixel transferred onto a wax stabilized membrane is shown.

Another crucial point for successful rLIFT onto commercial sensors is the size of the SnO_2_ pixel. If the pixel is too large a short circuit between the heater contact and the IDT may occur. Therefore, to avoid a cross resistance between the heater and the electrodes, the pixel size is decreased to about 300 μm in diameter, which is slightly smaller than the IDT structure (see Fig. 6). An investigation by optical microscopy (Fig. 6) confirms that the transferred material is only deposited on the IDT.

In addition to printing SnO_2_ pixels, precursor films containing Pd are prepared and transferred, due to the fact that according to literature^27^ 0.5% Pd should increase the sensitivity towards methane.

**Figure 7:**
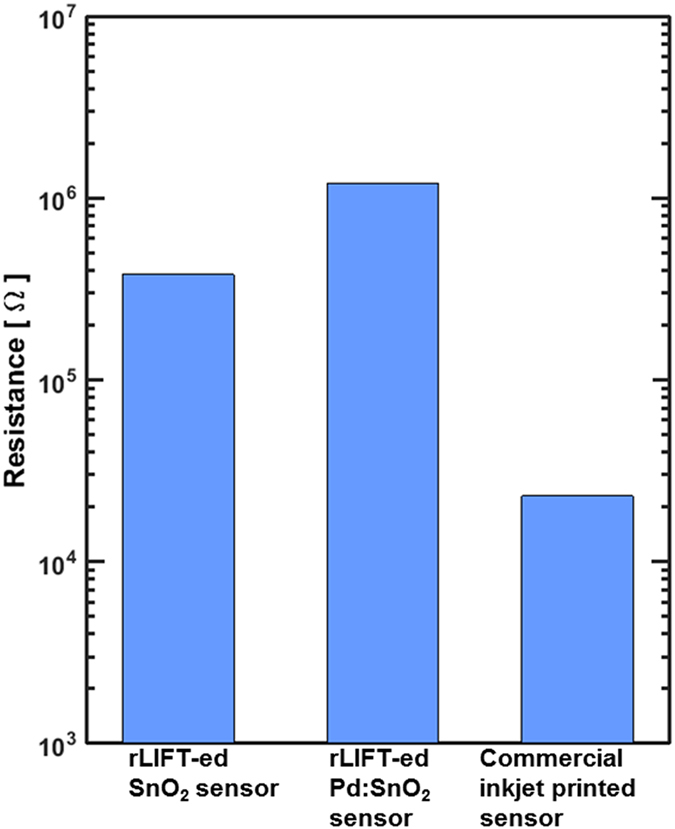
Base resistance at 350 °C of the LIFTed SnO_2_ sensors, Pt doped SnO_2_ sensors, and commercial inkjet printed sensors.

In order to evaluate rLIFT as an alternative method for the fabrication of micro-sized sensing devices, the SnO_2_ and Pd:SnO_2_ sensors fabricated by rLIFT are compared with the commercial inkjet printed sensors. In Fig. 7 the measured resistance of the SnO_2_ and Pd:SnO_2_ laser deposited sensors, and also the resistance of the commercial inkjet printed sensors are presented. The resistance of all rLIFT printed sensors is of at least one order of magnitude higher than the resistance of commercial inkjet printed sensors. This is most probably due to the much thinner SnO_2_ layer but this presents no problem as the standard read out electronics of such a sensor can be easily adapted to these higher resistances.

**Figure 8:**
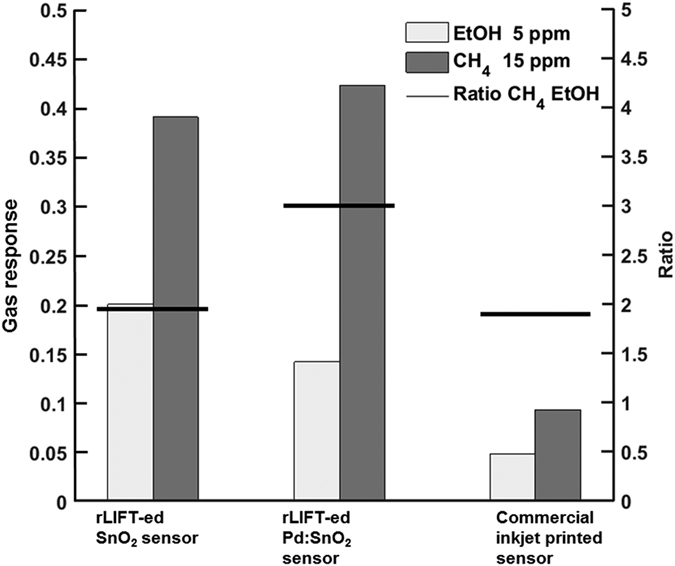
Sensor responses to 5 ppm of ethanol (at 350 °C) and 15 ppm of methane (at 500 °C). The black line indicates the ratio (S[CH_4_]/S[C_2_H_5_OH]) between the two sensitivities to visualize the influence of Pd doping.

Furthermore, the performance of the rLIFTed sensors is compared to the performance of the commercial inkjet printed sensors by measuring the response of the sensors to different concentrations of analytes (ethanol and methane). The response of the different gas sensors to 5 ppm C_2_H_5_OH and 15 ppm of methane is shown in Fig. 8. In addition, the corresponding real-time sensor responses to 5 ppm C_2_H_5_OH and 15 ppm of methane are shown in Figs SI1 and SI2. The overall picture shows a very high sensitivity for the rLIFT printed sensors compared to the commercial inkjet printed sensors. The response towards C_2_H_5_OH of the printed SnO_2_ sensors (S = 0.2) is slightly higher than the response of the Pd:SnO_2_ rLIFT-ed sensors (S = 0.15), and four times higher than for the commercial inkjet printed sensors (S = 0.05). This is in good agreement with the fact that Pd should enhance the response only towards CH_4_. The response enhancement towards methane by the Pd containing sensors could be ascribed to the spillover of dissociated methane molecules, i.e. CH_4_ dissociates on the Pd surface to a methyl group and a hydrogen adatom^35^, which then reacts with adsorbed oxygen to produce water (rapidly desorbed from the surface as the sensors are operated at 500 °C) and free electrons. [35 and refs therein].

On the other hand, the Pd doped sensors show the highest response towards CH_4_ (S > 0.4) when compared to the SnO_2_ rLIFT-ed sensors (S = 0.39), and to the response of only 0.09 for the commercial inkjet printed sensor. To see the influence of the dopant, the ratio between the response towards C_2_H_5_OH and CH_4_ is calculated. The horizontal lines in Fig. 8 indicate the ratio between the two analyte responses, defined as S[CH_4_]/S[C_2_H_5_OH]. This ratio does not indicate how well a sensor works. A change in this number just indicates for which analyte a certain sensor gives a better analyte response. For the undoped samples as well as for the commercial inkjet printed sensor the ratio is below 2. This means that these samples are better for detecting C_2_H_5_OH than CH_4_. On the other hand, the Pd doped samples show a ratio which is for all samples higher than 2.5 indicating that Pd enhanced the sensitivity towards CH_4_.

## Methods

### LIFT experimental setup

In order to carry out the rLIFT experiments a XeCl excimer laser with an emission wavelength of 308 nm (Lambda Physik, 30 ns pulse length) is used for the transfer. A homogeneous part of the laser beam is cut out, resulting in an almost flat top energy beam profile for the LIFT experiments. The pulsed XeCl laser is operated at a repetition rate of 1 Hz and is controlled by a mechanical shutter. The laser beam intensity is controlled with a computer controlled attenuator plate. The pulse energy is measured by a pyroelectric energy meter (Gentec QE 50) placed at the end of the beam line and the average of 50 pulses is used to determine the laser fluence. A circular 4 mm diameter aperture is used and demagnified four times to ablate pixels of 1 mm diameter. Laser fluences between 200 and 400 mJ/cm^2^ are used for the transfers.

The donor and the receiver are placed on a computer controlled translation stage while the laser irradiates the donor from the backside. The transfer of donor materials is investigated in the case when the donor and the receiver plates are placed at a distance of approximately 10 μm. All the transfers are carried out under ambient conditions (atmospheric pressure and 23 °C). A scheme of the LIFT experimental process is shown in Fig. 1.

### Donor materials

In this work a new approach to LIFT is used, which aims at partially decomposing a precursor material to form SnO_2_. In particular, metal complex precursors, i.e. SnCl_2_(acac)_2_ absorb UV light and decompose partially during UV laser transfer.

SnCl_2_(acac)_2_ is synthesized according to the procedure described in^36^,^37^. SnCl_2_ is mixed either with acetylacetone and HCl or with acetone. 1w% Triton X-100 is added to the final solution in order to improve the wettability when spin coating on the quartz substrates. The solution is filtered with a 1 μm filter and spin coated at 2500 rpm resulting in thin films with a thickness of approximately 900 nm.

Doping SnO_2_ with certain metals may increase the sensor response and selectivity towards different analytes such as CH_4_^38^. In this case, palladium doping is chosen as a Pd content of approximately 0.5% is expected to increase the sensor response towards CH_4_ detection. A commercially available Pd metal precursor solution (i.e. Pd(acac)_2_) is purchased from Sigma Aldrich and 0.5% wt of Pd(acac)_2_ is added to the SnCl_2_(acac)_2_ solution applied for spin coating.

### Optical microscopy

The transferred pixels as well as the donor films prior to ablation are investigated by optical microscopy. The images are acquired with a Zeiss Axioplan microscope coupled with a Leica digital camera. For low magnification images an Olympus SZH 10 Research Stereo microscope with a Stingray F145C CCD camera is used.

### Receiver substrates - Sensor pads and commercial sensors

For the functional characterization of the rLIFT printed materials, sensor-like pads are designed using the same interdigitated electrode (IDT) structure as the commercial sensors (MICROSENS gas sensor, MSGS 3000). On the sensor-like pads the structure is not free standing and therefore no stabilization of a membrane structure (as in the case of the commercial sensors) is required.

The IDT structures are implemented on borax glass substrates. The electrodes on top are sputtered using first a 20 nm chromium interlayer with 100 nm platinum on top.

The main sensing device of the commercial MSGS 3000 sensor, as well as of other commercially available SnO_2_ sensors, is based on silicon microstructures. The main sensor chip is a rectangular shaped structure with a side length of 1900 × 1700 μm. The part underneath the IDT supporting the SnO_2_ is a thin membrane. The thin membrane (900–1000 nm thickness) has the role to maintain a low thermal mass and to reduce the heat conductivity towards the sides.

Underneath the membrane is a heater which brings the SnO_2_ to temperatures in the range of 300 °C to 600 °C. The top part of the membrane is electrically insulating SiO_2_ with sputtered Pt IDT electrodes on top. The electrodes are coated with SnO_2_ and the resistance between the metal finger electrodes is measured.

For the commercial MSGS sensor the SnO_2_ coating is carried out by ink-jet printing. In the case of the LIFT experiments uncoated commercial MSGS sensors are used (and compared to the inkjet printed MSGS sensors).

### Thermal treatment of the rLIFT-ed material on the sensor

The rLIFT printed SnO_2_ were submitted to the same thermal treatment as the commercial sensors for reasons of comparison^39^. The lifted sensor pads are heated for 24 h at 350 °C followed by 6 h at 500 °C and 6 h at 600 °C in a stream of 1 l/min of synthetic air (SA) containing 20% O_2_ and 80% N_2_.

### Gas sensing setup

Measuring and analyzing the performance of the LIFT printed sensor-like structures is carried out in a controlled atmosphere with the possibility of adding analytes in the ppm range, while heating the sensor-like structures up to 350 °C for ethanol and 500 °C for methane. The sensor-like structures are mounted on an alumina (Al_2_O_3_) block containing a K-type thermocouple for temperature measurements. The sensor-like samples are contacted electrically by two metal clamps, on the side pressing a graphite rode onto the Pt-electrodes reaching a total contact resistance of less than 50 Ω. Graphite rods are needed to prevent the Pt-electrodes to be scratched off.

Temperature measurements and resistance measurements are acquired by a computer controlled (LabView) setup using a Keithley 2400 source meter and Keithley 2000 multi meter respectively. The alumina block with the sensor on top is placed in a tube furnace with a constant gas supply.

The main gas supply is dry synthetic air (SA) with 80% N_2_ and 20% O_2_, with a standard gas flow of 5 l/min. For humidifying the air, the main gas flow is bubbled through a flask containing few deciliters of distilled water.

The test analytes, i.e. either ethanol or methane, are added (separate experiments) with a low flow rate (0.01 to 0.1 l/min) to the main gas flow just after the water containing flask. Analyte concentration in the ppm range, i.e. 10 ppm for ethanol and 30 ppm for methane, could be achieved.

## Conclusions

The results shown in this paper reveal that reactive laser induced forward transfer is an appropriate technique for fabricating efficient SnO_2_ and Pd:SnO_2_ based sensors. The base resistance of the rLIFTed sensors was measured in dry as well as in humid air. The SnO_2_ transferred pixels have a resistance in a suitable range for sensing applications. Analyzing the sensor responses towards C_2_H_5_OH revealed that the laser transferred SnO_2_ pixels have a very high response and most of the sensor-like pads were close to saturation even when exposed to 10 ppm of C_2_H_5_OH.

The rLIFTed sensor-like pads are stable over long time periods. Repeating the measurements with the same sample after one year showed that LIFT fabricated SnO_2_ sensors have a very low degradation.

Another key analyte for SnO_2_ gas sensors is the detection of CH_4_. It has been found that 50 ppm CH_4_ could be detected in dry air.

To understand the influence of the metal complex precursor deposition method on the sensing performance of the sensors, precursor materials were drop casted onto sensor-like pads. The sensor response measurements showed a much lower sensitivity in comparison with the rLIFT printed sensor-like pads. Therefore, we can assume that the rLIFT process is the key factor to achieve SnO_2_ pixels with high gas responses.

The high response towards C_2_H_5_OH and CH_4_ determined on the sensor-like pads is confirmed. The sensitivity of the SnO_2_ laser printed sensors shows four times better performance than the commercial inkjet printed sensors. Pd doped SnO_2_ has been printed and the response towards CH_4_ is improved reaching a four times better response than the commercial inkjet printed sensor. This reveals that rLIFT is a competitive alternative to commercial printing techniques, i.e. sputtering, inkjet printing for fabricating high performance sensors.

These results represent important steps to expanding the capability of rLIFT as a processing method for the fabrication of SnO_2_ and decorated SnO_2_ micro-devices.

## Additional Information

**How to cite this article**: Palla Papavlu, A. *et al.* Highly sensitive SnO_2_ sensor via reactive laser-induced transfer. *Sci. Rep.* **6**, 25144; doi: 10.1038/srep25144 (2016).

## Supplementary Material

Supplementary Information
