# Supplementary material for: Highly sensitive SnO2 sensor via reactive laser-induced transfer
Source: Sci Rep. 2016 Apr 27;6:25144. doi: 10.1038/srep25144 (PMC4846859; doi:10.1038/srep25144)
Supplement: Supplementary Information [file srep25144-s1.doc]

Supplementary Information

Highly sensitive SnO_2_ sensor via reactive laser-induced transfer

Alexandra Palla Papavlu^1,2,&^, Thomas Mattle^1,*^, Sandra Temmel^1^, Ulrike Lehmann^3,4^, Andreas Hintennach^1,#^, Alain Grisel^3,4^, Alexander Wokaun^1^, Thomas Lippert^1,5,&^

^1^ Paul Scherrer Institut, Energy and Environment Research Division, 5232 Villigen- PSI, Switzerland

^2^ National Institute for Lasers, Plasma, and Radiation Physics, Lasers Department, Atomistilor 409, 077125, Magurele, Romania

^3^ Microsens SA, Rue de la Maladière 71c, CH-2002 Neuchâtel, Switzerland

^4^ EPFL Innovation Park, Building D, CH-1015 Lausanne, Switzerland

^5^ Department of Chemistry and Applied Biosciences, Laboratory of Inorganic Chemistry, ETH Zürich, CH-8093 Zürich, Switzerland

& Corresponding authors: [thomas.lippert@psi.ch](mailto:thomas.lippert@psi.ch) and [alexandra.palla-papavlu@psi.ch](mailto:alexandra.palla-papavlu@psi.ch)

* Current address: Geberit International AG, Schachenstrasse 77, 8645 Jona, Switzerland

# Current address: Electrochemical Layers, Daimler AG (Mercedes-Benz Cars) HPC H152, 70176 Stuttgart, Germany


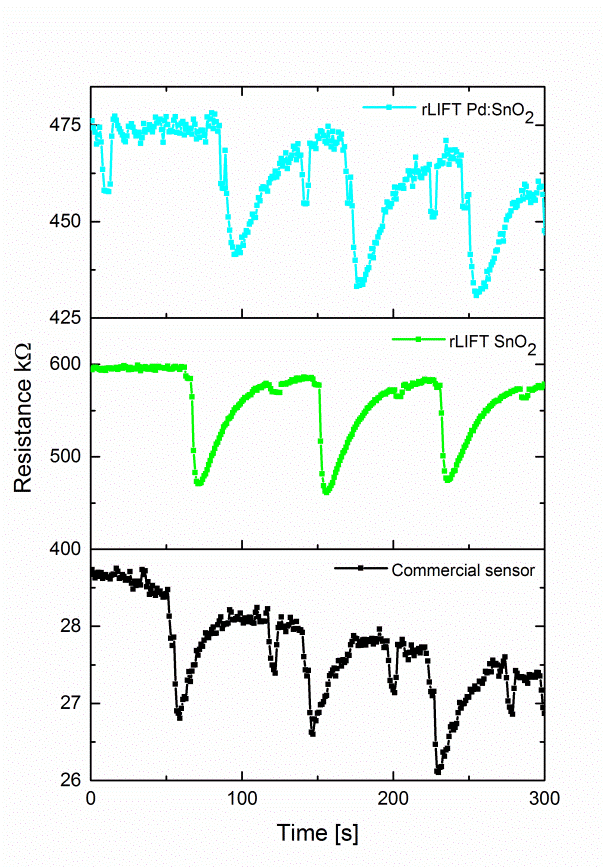


**Figure SI 1** Real time response curves of different sensors i.e. commercial sensors fabricated by inkjet printing, SnO_2_ and Pd:SnO_2_ based sensors fabricated by rLIFT when exposed to 5 ppm of ethanol.

**
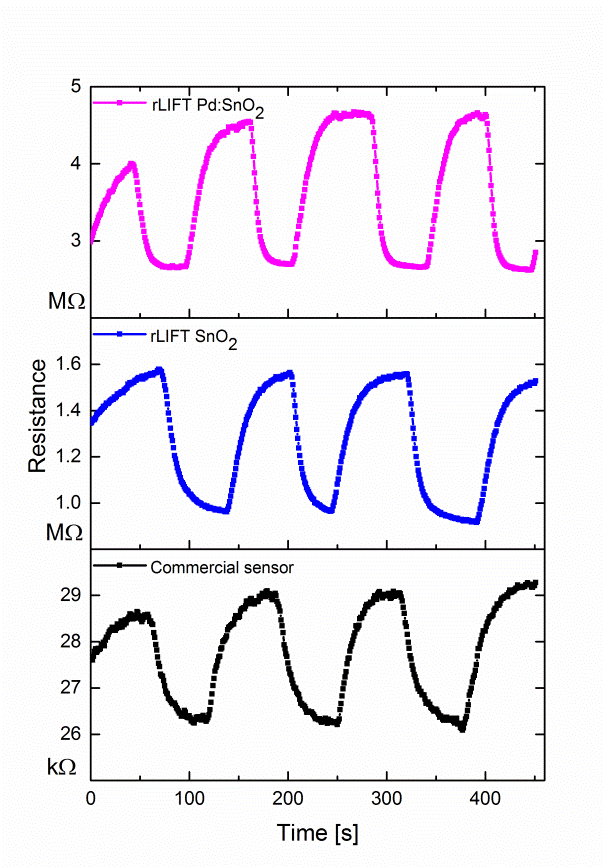
**

**Figure SI 2** Real time response curves of different sensors i.e. commercial sensors fabricated by inkjet printing, SnO_2_ and Pd:SnO_2_ based sensors fabricated by rLIFT when exposed to 15 ppm of methane.


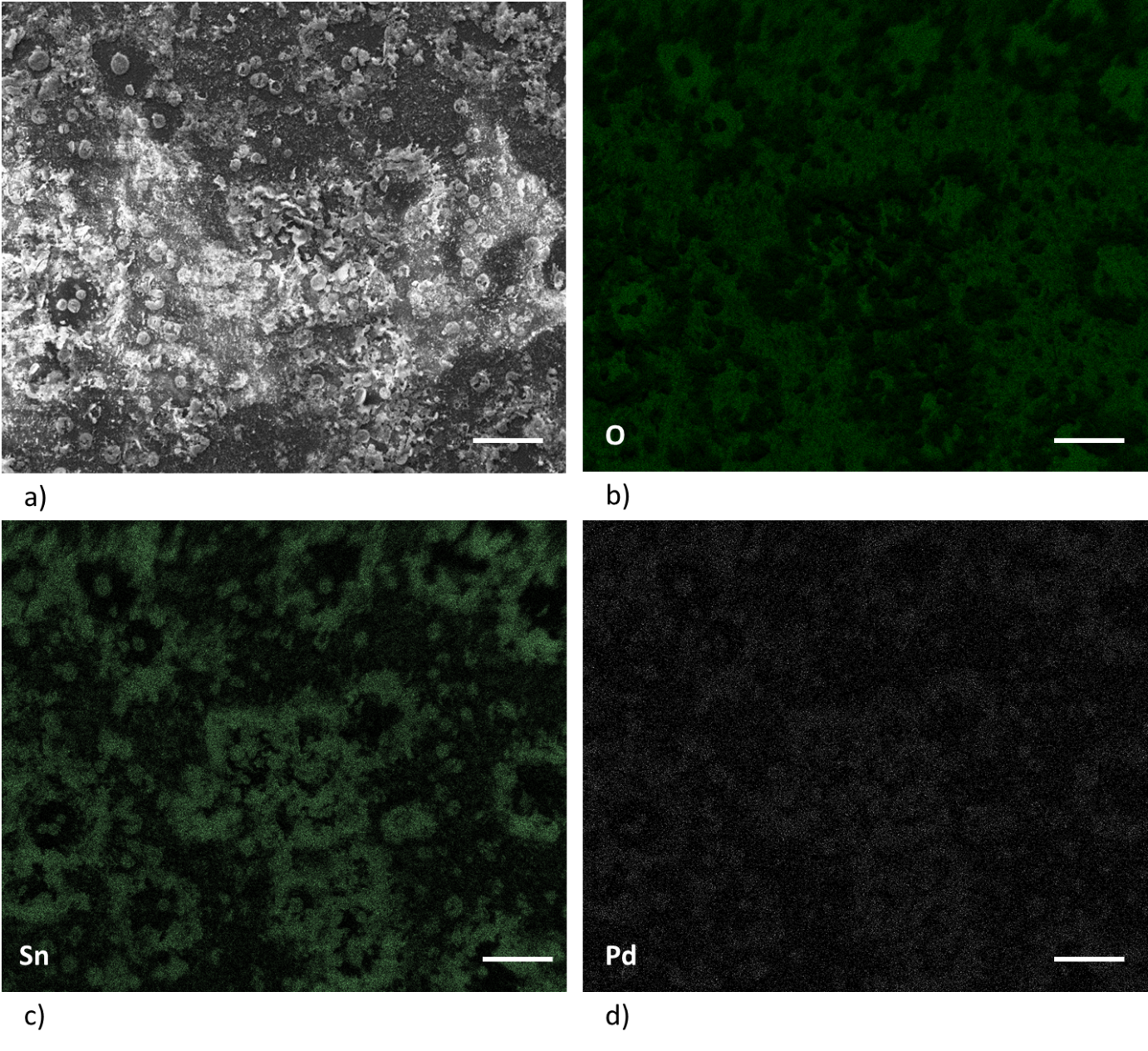


**Figure SI 3** a) Scanning electron microscope (SEM) image of Pd:SnO_2_ pixel transferred at laser fluence and the corresponding EDS elemental maps of (b) O, (c) Sn, and (d) Pd. The scale bars in the electron micrographs correspond to 50 µm.

As revealed by energy dispersive X-ray spectroscopy (EDS) (Figure SI 3), Pd is dispersed homogeneously on the SnO_2_ surface.

The chemical states of Pd and Sn in the transferred pixels were analyzed by x-ray photoelectron spectroscopy (XPS). The XPS spectra were recorded using a SPECS XPS spectrometer based on Phoibos 150 MCD electron energy analyzer operated in constant energy mode. The Al Kα line, centered at 1486,74 eV was used for monochromatic X-ray excitation. High resolution spectra were acquired over ranges of 20 eV, at 20 eV pass energy and energy resolution of 0.05 eV. The analysis was carried out in 10^-9^ mbar ultra-high vacuum conditions. The recorded spectra were processed using SDP version 7.0 software.


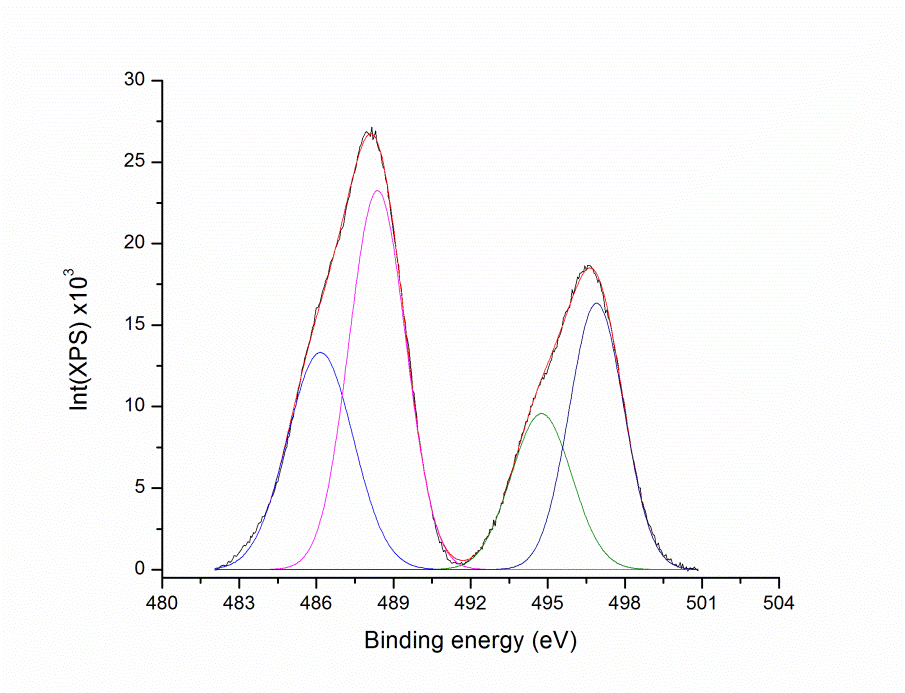


**Figure SI 4 a)** The Sn 3d XPS spectra of the Pd-decorated SnO_2_ pixels transferred by LIFT.


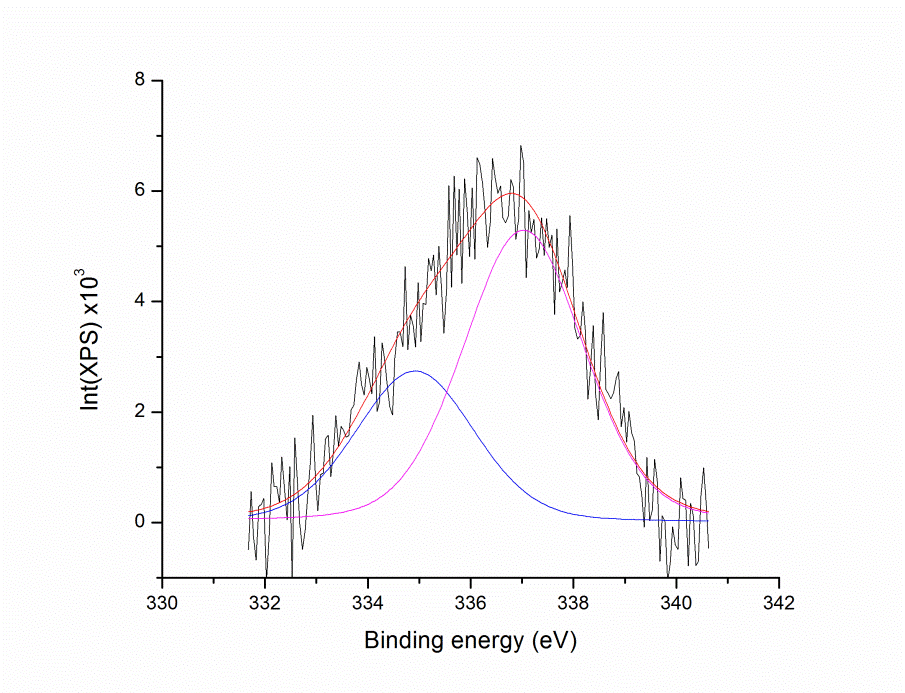


**Figure SI 4 b)** XPS spectra of Pd 3d for the Pd-decorated SnO_2_ pixels transferred by LIFT.

From Fig. SI 4 the following components are identified: metallic Pd (Pd 3d_5_ at 334,9 eV) and Pd oxide (PdO at 337 eV) with binding energies in good agreement with those reported elsewhere [1,2]. The XPS measurements show that the Pd:SnO_2_ pixels contain both metallic Pd and Pd monoxide, with PdO being dominant, as expected from the applied temperature [1-3].

The PdO is most probably obtained during the post-treatment of the sensors, i.e. during the annealing step.

References

[1] Cao, X., Cao, L., Yao, W., Ye, X., Structural characterization of Pd-doped SnO2 thin films using XPS, *Surf Interface Anal* **24**, 662-666 (1996).

[2] Kappler, J., Barsan, N., Weimar, U., Dieguez, A., Alay, J.L., Romano-Rodriguez, A., Morante, J.R., Gopel, W., Correlation between XPS, Raman and TEM measurements and the gas sensitivity of Pt and Pd doped SnO2 based gas sensors, *Fresenius J Anal Chem* **361**, 110-114 (1998).

[3] Batzill, M., Diebold, U., The surface and materials science of tin oxide, *Progress in Surface Science* **79**, 47-154 (2005).
